# US-like isolates of porcine epidemic diarrhea virus from Japanese outbreaks between 2013 and 2014

**DOI:** 10.1186/s40064-015-1552-z

**Published:** 2015-12-02

**Authors:** Nguyen Van Diep, Junzo Norimine, Masuo Sueyoshi, Nguyen Thi Lan, Takuya Hirai, Ryoji Yamaguchi

**Affiliations:** Laboratory of Veterinary Pathology, Department of Veterinary, Faculty of Agriculture, University of Miyazaki, 1-1 Gakuenkibanadai-Nishi, Miyazaki, 889-2192 Japan; Faculty of Veterinary Medicine, Vietnam National University of Agriculture, Hanoi, Vietnam

**Keywords:** Porcine epidemic diarrhea virus, PEDV Japan, PED, Partial S gene, ORF3

## Abstract

Since late 2013, outbreaks of porcine epidemic diarrhea virus (PEDV) have reemerged in Japan. In the present study, we observed a high detection rate of PEDV, with 72.5 % (148/204) of diarrhea samples (suckling, weaned, and sows) and 88.5 % (77/87) of farms experiencing acute diarrhea found to be positive for PEDV by reverse transcription PCR. Sequencing and phylogenic analyses of the partial spike gene and ORF3 of PEDV demonstrated that all prevailing Japanese PEDV isolates belonged to novel genotypes that differed from previously reported strains and the two PEDV vaccine strains currently being used in Japan. Sequence and phylogenetic analysis revealed prevailing PEDV isolates in Japan had the greatest genetic similarity to US isolates and were not vaccine-related. Unlike vaccine strains, all prevailing field PEDV isolates in Japan were found to have a number of amino acid differences in the neutralizing epitope domain, COE, which may affect antigenicity and vaccine efficacy. The present study indicates recent PEDV isolates may have been introduced into Japan from overseas and highlights the urgent requirement of novel vaccines for controlling PEDV outbreaks in Japan.

## Background

Porcine epidemic diarrhea (PED) is a highly contagious and devastating viral enteric disease characterized by vomiting, acute onset of severe watery diarrhea, and dehydration. PED has a high infectivity and a particularly significant mortality in piglets (Pensaert and de Bouck 1978). The porcine epidemic diarrhea virus (PEDV), an enveloped, single-stranded RNA virus belonging to the *Alphacoronavirus* genus of the *Coronaviridae* family, is responsible for PED. The PEDV genome is approximately 28 Kb in length and is composed of seven open reading frames (ORF) that encode four structural proteins, namely, spike (S), envelope (E), membrane (M), and nucleocapsid (N), and three major non-structural proteins, including replicases 1a and 1b, and ORF3 (Song and Park 2012). Of the structural protein, the PEDV S protein plays a pivotal role in regulating interactions with specific host cell receptors to mediate viral attachment and entry. Moreover, the S protein is associated with the induction of host neutralizing antibodies, growth adaptation in vitro, and attenuation of virulence in vivo (Song and Park 2012). Thus, study of the S glycoprotein has been essential in understanding the genetic relationships between PEDV strains, the epidemiological status of PEDV in the field, and the development of vaccines (Song and Park 2012; Chen et al. 2012); (Temeeyasen et al. 2014).

In addition to the S glycoprotein gene, the ORF3 gene has received a large amount of attention in the aspect of PEDV virulence. ORF3 gene plays a role in encoding an ion channel protein (Wang et al. 2012) and it has been suggested to be an important determinant for virulence of this virus (Song and Park 2012). The virulence of PED can be reduced by altering the ORF3 gene through cell culture adaptation (Park et al. 2008), and variation in ORF3 was reported to be associated with viral attenuation in the natural host (Song et al. 2003). Also, vaccine-derived isolates with unique continuous deletions of 49 and 51 ORF3 nucleotides have been confirmed (Chen et al. 2010; Park et al. 2008). Therefore, these unique deletions in the ORF3 gene can be used to differentiate between field and attenuated vaccine strains. Moreover, ORF3 gene variation may represent a useful tool in molecular epidemiological studies of PEDV (Park et al. 2008, 2011; Song et al. 2003; Chen et al. 2010).

In Japan, the first outbreak of PED-like disease was reported in late 1982 and early 1983 (Kusanagi et al. 1992; Takahashi et al. 1983), and was followed by pandemics between late 1993 and 1996 (Sueyoshi et al. 1995; Tsuda 1997). Afterwards, there have been sporadic PED outbreaks in intervals of several years. Since late 2013, numerous diarrhea epidemics, suspected to be caused by PED, have occurred in pigs throughout Japan. These epidemics were characterized by severe diarrhea, dehydration, and vomiting in pigs of all ages. Mortality rates were particularly high among suckling pigs. Up to the end of August 2014, more than 410,000 of 1,286,000 pigs from 817 infected farms have died of PED in Japan based from the report of Ministry of Agriculture, Forestry and Fisheries (MAFF) (http://www.maff.go.jp). However, there have been few studies investigating the re-emergence of PEDV in Japan. This study aimed to evaluate the genetic characteristics and molecular epidemiology of the emergent Japanese PEDV isolates using genome analysis and phylogenetic analysis of the partial S gene and ORF3.

## Results

### PEDV detection

A total of 72.5 % (148 of 204) of samples (suckling, weaned, and sows) from 77 pig farms (88.5 %) experiencing acute diarrhea in six prefectures were found to be positive for PEDV by RT-PCR. PEDV-positive samples were identified from the following prefectures: Miyazaki (n = 107), Kagoshima (n = 9), Aichi (n = 15), Akita (n = 1), Hokkaido = 7), and Aomori (n = 9). To investigate the heterogeneity of the recent Japanese isolates and their genetic relationship with modified live vaccines, in addition to 2 PEDV vaccine strains (P5-V and 96-P4C6) used in Japan, representative isolates were selected for sequencing of the partial S gene and full ORF3 gene.

### Sequence and phylogenetic analysis of the partial S gene

**Fig. 1:**
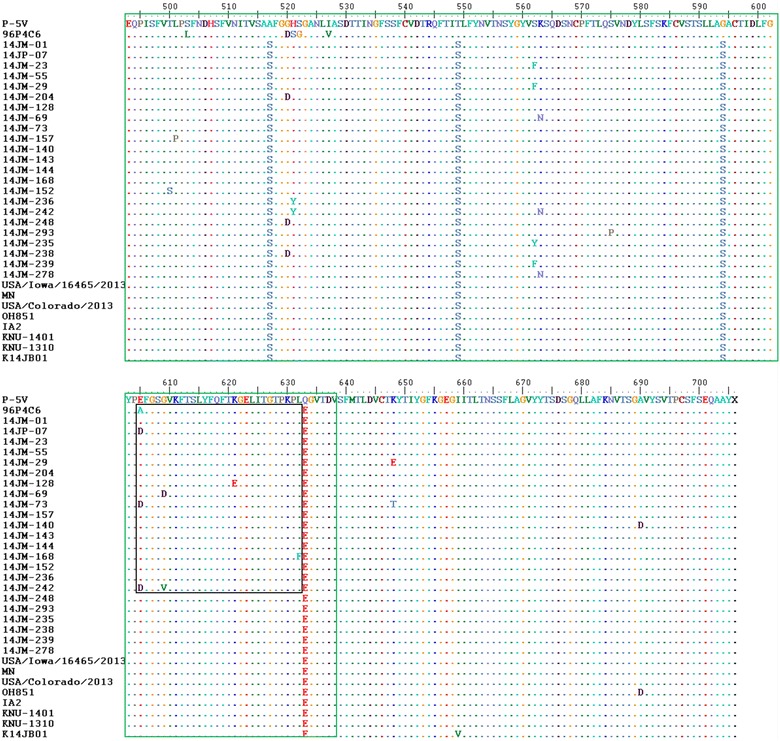
Comparison of deduced amino acid sequence alignment of the partial S gene of two vaccine strains, 23 Japanese fields PEDV isolates, US strains, and US like-strains from South Korean. *Dash* (.) reveals the amino acid identity of isolates compared with vaccine strain P5-V. The *green box* shows the COE domain. The *black box* indicates the variable site on the COE domain

The partial S gene, including the CO-26K equivalent (COE) domain, of 80 PEDV samples from 69 PEDV-infected farms were amplified, purified, and sequenced. The partial S sequences were aligned at nucleotides 1477–2116 (amino acids 493–705) of the full S gene. Identical nucleotide sequences were distinguished and excluded, resulting in the identification of 23 individual sequences from the total of 80 field PEDV isolates (Table 1). However, sequencing revealed high genetic variation between nucleotides 1815 and 1944 (amino acid residues 605–648). A total of 20 nucleotide substitutions were detected, leading to 13 amino acid changes, within the partial S gene (Fig. 1).

**Table 1:** Names and accession numbers of vaccine strains and Japanese field isolates with distinct sequences of the partial S gene and ORF3 gene in this study

No.	Name of isolates	Age group	Sample origin	Collection time	Geographic origin	Partial S gene	ORF3 gene
1	14JM-01^a^	Suckling	Small intestine	2014/March	Miyazaki	KT968486	KT968511
2	14JM-07	Suckling	Small intestine	2014/April	Miyazaki	KT968487	*
3	14JM-23^b^	Suckling	Small intestine	2014/April	Aichi	KT968488	*
4	14JM-29	Suckling	Feces	2014/April	Aichi	KT968490	*
5	14JM-55	Suckling	Feces	2014/April	Akita	KT968489	*
6	14JM-69	Suckling	Feces	2014/April	Miyazaki	KT968493	*
7	14JM-73	Suckling	Feces	2014/April	Miyazaki	KT968494	*
8	14JM-128^d^	Suckling	Intestinal content	2013/December	Miyazaki	KT968492	*
9	14JM-140^f^	Suckling	Intestinal content	2014/March	Miyazaki	KT968496	*
10	14JM-143	Suckling	Intestinal content	2014/February	Miyazaki	KT968497	*
11	14JM-144	Suckling	Intestinal content	2014/March	Miyazaki	KT968498	*
12	14JM-152^g^	Suckling	Small intestine	2014/March	Miyazaki	KT968500	*
13	14JM-157^e^	Suckling	Feces	2014/May	Aichi	KT968495	*
14	14JM-168	Suckling	Feces	2014/May	Aomori	KT968499	*
15	14JM-204^c^	Suckling	Feces	2014/June	Hokkaido	KT968491	KT968513
16	14JM-235	Suckling	Feces	2014/June	Miyazaki	KT968505	*
17	14JM-236	Suckling	Feces	2014/July	Miyazaki	KT968501	*
18	14JM-238	Suckling	Feces	2014/June	Miyazaki	KT968506	KT968514
19	14JM-239	Suckling	Feces	2014/July	Miyazaki	KT968507	*
20	14JM-242	Suckling	Feces	2014/May	Miyazaki	KT968502	*
21	14JM-248	Suckling	Feces	2014/January	Miyazaki	KT968503	*
22	14JM-278	Suckling	Feces	2014/February	Miyazaki	KT968508	KT968515
23	14JM-293^h^	Suckling	Feces	2013/December	Kagoshima	KT968504	*
24	14JM-40	Suckling	Feces	2014/April	Hokkaido		KT968512
25	14JM-295^a^	Suckling	Feces	2014/January	Kagoshima		KT968516
26	14JM-02^a^	Suckling	Small intestine	2014/March	Miyazaki		
27	14JM-12^a^	Suckling	Small intestine	2014/April	Miyazaki		
28	14JM-19^a^	Sow	Feces	2014/April	Miyazaki		
29	14JM-34^a^	Sow	Feces	2014/April	Aichi		
30	14JM-46^a^	Suckling	Intestinal content	2014/April	Miyazaki		
31	14JM-48^a^	Suckling	Intestinal content	2014/April	Miyazaki		
32	14JM-63^a^	Sow	Feces	2014/April	Miyazaki		
33	14JM-65^a^	Sow	Feces	2014/April	Miyazaki		
34	14JM-117^a^	Suckling	Small intestine	2014/April	Miyazaki		
35	14JM-118^a^	Suckling	Small intestine	2014/March	Miyazaki		
36	14JM-119^a^	Suckling	Feces	2014/April	Miyazaki		
37	14JM-120^a^	Suckling	Feces	2014/April	Miyazaki		
38	14JM-121^a^	Suckling	Feces	2014/May	Miyazaki		
39	14JM-122^a^	Suckling	Feces	2014/March	Miyazaki		
40	14JM-123^a^	Suckling	Feces	2014/March	Miyazaki		
41	14JM-124^a^	Suckling	Feces	2014/March	Miyazaki		
42	14JM-125^a^	Suckling	Feces	2014/March	Miyazaki		
43	14JM-126^a^	Suckling	Feces	2014/March	Miyazaki		
44	14JM-127^a^	Suckling	Feces	2013/December	Miyazaki		
45	14JM-129^a^	Suckling	Feces	2014/January	Aichi		
46	14JM-130^a^	Suckling	Feces	2014/January	Aichi		
47	14JM-131^a^	Suckling	Feces	2014/February	Aichi		
48	14JM-132^a^	Suckling	Feces	2014/January	Aichi		
49	14JM-133^a^	Suckling	Feces	2014/January	Aichi		
50	14JM-134^a^	Suckling	Feces	2014/February	Miyazaki		
51	14JM-135^a^	Suckling	Feces	2014/January	Miyazaki		
52	14JM-136^a^	Suckling	Feces	2013/December	Miyazaki		
53	14JM-137^a^	Suckling	Feces	2014/January	Miyazaki		
54	14JM-139^a^	Suckling	Feces	2014/March	Miyazaki		
55	14JM-141^a^	Suckling	Feces	2014/April	Miyazaki		
56	14JM-142^a^	Suckling	Feces	2014/March	Miyazaki		
57	14JM-145^a^	Suckling	Feces	2014/April	Miyazaki		
58	14JM-146^a^	Suckling	Feces	2014/April	Miyazaki		
59	14JM-147^a^	Suckling	Feces	2014/May	Miyazaki		
60	14JM-149^a^	Suckling	Feces	2014/April	Miyazaki		
61	14JM-150^a^	Suckling	Feces	2014/March	Miyazaki		
62	14JM-151^a^	Suckling	Feces	2014/March	Miyazaki		
63	14JM-153^a^	Suckling	Feces	2014/March	Aomori		
64	14JM-154^a^	Suckling	Feces	2014/April	Aomori		
65	14JM-174^a^	Suckling	Feces	2014/May	Aomori		*
66	14JM-24^b^	Suckling	Feces	2014/April	Aichi		
67	14JM-37^c^	Suckling	Feces	2014/April	Hokkaido		*
68	14JM-45^c^	Suckling	Feces	2014/April	Hokkaido		
69	14JM-203^c^	Suckling	Feces	2014/June	Hokkaido		
70	14JM-56^d^	Suckling	Feces	2014/April	Aomori		
71	14JM-60^e^	Suckling	Feces	2014/April	Aomori		
72	14JM-138^f^	Suckling	Feces	2014/March	Miyazaki		
73	14JM-162^b^	Suckling	Feces	2014/May	Aichi		
74	14JM-179^e^	Suckling	Feces	2014/May	Aomori		
75	14JM-200^g^	Suckling	Feces	2014/May	Miyazaki		
76	14JM-210^b^	Suckling	Intestinal content	2014/July	Aichi		
77	14JM-226^h^	Suckling	Feces	2014/July	Kagoshima		*
78	14JM-229^h^	Suckling	Intestinal	2014/July	Kagoshima		
79	14JM-230^h^	Suckling	Intestinal	2014/July	Kagoshima		
80	14JM-252^g^	Suckling	Feces	2014/March	Miyazaki		
81	P5-V		Nisseiken Co.			KT968509	KT968517
82	P6P4C6		Kakatsuken Co.			KT968510	KT968518

* PEDV isolates that the sequences of ORF3 gene was identical to that of the isolate 14JM-01

“a, b, c, d, e, f, g, h”: PEDV isolates having the same the letter have the same sequence of the partial S gene

The COE domain (amino acids 499–638) of the S protein consists of 140 aa and contains epitopes that are capable of inducing PEDV-neutralizing antibodies (Chang et al. 2002). Compared to the two vaccine strains (P-5V and 96-P4C6), all Japanese field strains had 3 different amino acids at positions 517 (A → S), 549 (T → S), and 594 (G → S) within the COE domain. Furthermore, differences in amino acids were found at the following 10 sites within the COE domain of the S protein: 500 (T → S), 501 (L → P), 521 (H → Y or S → Y), 562 (S → F or S → Y), 563 (K → N), 575 (S → P), 605 (E → D or A → D), 609 (G → D or G → V), 621 (K → E), and 632 (L → F) as shown in Fig. 1.

To investigate the heterogeneity of prevailing PEDV strains in Japan, phylogenetic tree of 23 partial S genes of PEDV field strains and two vaccine strains were constructed together with 4 previously reported Japanese PEDV strains (NK, KH, MK, 83P-5) and reference strains from other countries available in GenBank.

**Fig. 2:**
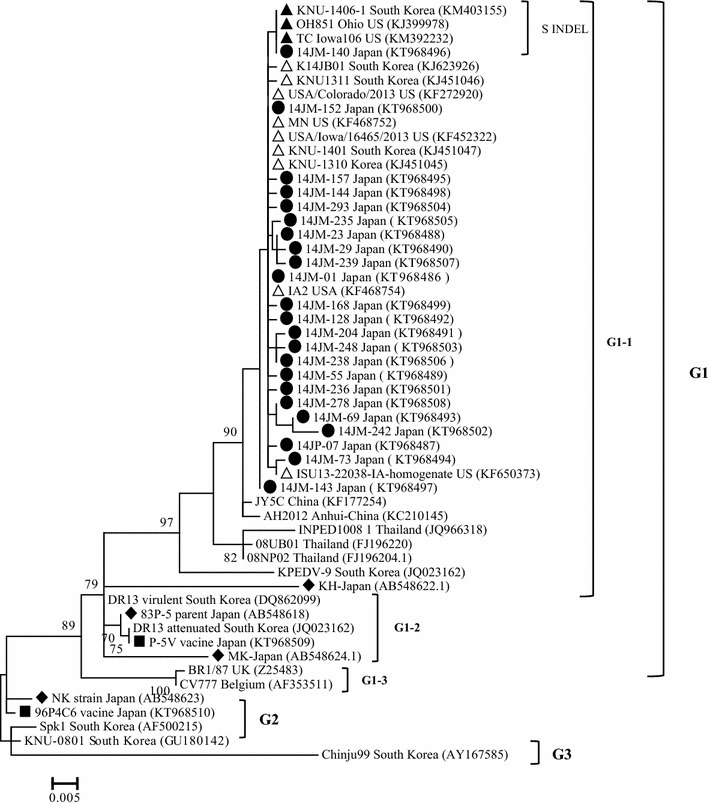
Phylogenetic analysis of the porcine epidemic diarrhea virus isolates based on the nucleotide sequences of the partials S genes. The tree was generated by the maximum likelihood method of the software MEGA v.6.05. Numbers at nodes represent the percentage of 1000 bootstrap replicates (values <70 are not shown). The *scale bar* indicates nucleotide substitution per site. The recent Japanese PEDV isolates in this study are marked by solid round symbols, the Japanese strains prior to 2013 are marked by *solid diamond symbols*, and the vaccine strains being used in Japan are marked by *solid square symbols*. The US strains and US-like strains are marked by *triangle hollow symbols*, while the S INDEL strains are marked by *solid triangle symbols*

Consistent with previous reports (Park et al. 2007; Puranaveja et al. 2009; Temeeyasen et al. 2014), phylogenetic tree based on partial S gene sequences in this study demonstrated that PEDV strains can be divided into three groups: G1, G2, and G3. Group G1 can be further divided into 3 subgroups: G1-1, G1-2 and G1-3 (Fig. 1). Notably, all field PEDV isolates circulating in Japan were found to cluster closest to isolates from the USA (USA/Iowa/16465/2013, OH851, IOWA106, MN, and IA2, USA/Colorado/2013, ISU13-22038-IA-homogenate), and US strain-like PEDV from South Korean (KNU-1401, KNU-1406-1, KNU-1310, KNU-1311) collected from 2013 to 2014 (Lee and Lee 2014; Lee et al. 2014); they were found cluster within the same subgroup G1-1 (Fig. 2). Further, isolate 14JM-140 clustered with S INDEL strains (OH851, IOW106, KNU-1406), forming a distinct minor branch within the cluster. On the other hand, vaccine strains P5-V and strain NK clustered within group G2, while the vaccine strain 96-P4C6 and old strains MK, KH, and 83P-5 belonged to subgroup G1-2. These results demonstrated distantly genetic relationships between field isolates and vaccine strains, in addition to previously reported PEDV strains in Japan.

Pairwise alignment of field Japanese PEDV isolates demonstrated high nucleotide and amino acid sequence identity between strains (99.1‒100.0 and 97.2‒100 %, respectively). Notably, 42 field PEDV isolates collected from 3 prefectures in this study had identical partial S gene sequence (100 % nucleotide homology). Japanese field isolates shared the highest DNA sequence identity (99.2–100 %) with American and South Korean strains, corresponding to 97.6‒100 % homology at the deduced amino acid level. Notably, the partial S gene sequences of 42 isolates (represented by the sequence of isolates 14JM-01) shared 100 % nucleotide identity with US strains (USA/Iowa/16465/2013, MN, IA2, and USA/Colorado/2013) and US like‒strains in South Korea (KNU-1401, HNU-1310). In contrast, field Japanese isolates had only 94.4‒97.2 % DNA sequence identity (94.0‒98.6 % amino acid homology) with PEDV strains prior to 2013 in Japan. The nucleotide identity of the vaccine strain P5-V with recent Japanese PEDV isolates (97.2–98.1 %) was higher than that of the vaccine strain 96-P4C6 (94.6‒95.6 %).

### Sequence and phylogenetic analysis of the ORF3 gene

To investigate the genetic relationship between recent Japanese field isolates, and modified live vaccine strains and reference strains, the nucleotide sequences of the ORF3 genes of 28 recent PEDV isolates and 2 vaccine samples (P5-V and 96-P4C6) were sequenced and analyzed (Table 1). Sequencing data revealed the ORF3 genes of all 28 PEDV samples were 675 bp in length and encoded a peptide 224 amino acid long, the same ORF3 gene length as the prototype, CV777. However, the ORF3 genes of P5-V and 96P4C6 were found to have 49-nt (at nt 244–292) and 4-nt (nt 413–416) deletions, respectively. The deletions in P5-V lead to a reading frame-shift and TAG terminator at 276nt while that of 96P4C6 resulted in a TGA terminator at 342nt. Thus, ORF3 of P5-V and 96P4C6 encoded truncated proteins of 91 and 143 amino acid, respectively. Twenty-one PEDV isolates collected from recent outbreaks in 5 prefectures (Miyazaki, Kagoshima, Aichi, Aomori, and Hokkaido) were found to have identical ORF3 gene sequence (represented by isolate 14JM-01). Identical sequences were excluded, resulting in 6 isolates for further analysis (Table 1). Sequence analysis revealed that the ORF3 genes of the Japanese field isolates were relatively well-conserved. Only 5 point substitutions were observed at nt 24, 51, 189, 302, and 501, with only the substitution at nt 302 resulting in a non-synonymous substitution of T to I at residue 100.

**Fig. 3:**
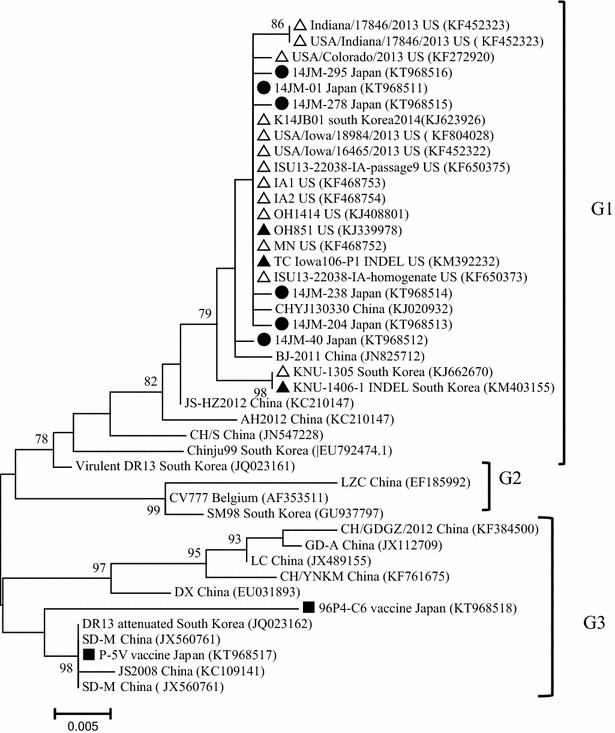
Phylogenetic analysis of the porcine epidemic diarrhea virus isolates based on the ORF3 of PEDV. The tree was generated by the maximum likelihood method of the software MEGA v.6.05. Numbers at nodes represent the percentage of 1000 bootstrap replicates (values <70 are not show). The *scale bar* indicates nucleotide substitution per site. The recent Japanese PEDV isolates in this study are marked by *solid round symbols* and the vaccine strains being used in Japan are marked by *solid square symbols*. The US strains and US-like strains are marked by *triangle hollow symbols*, while the S INDEL strains are marked by *solid triangle symbols*

Phylogenetic analyses revealed that, based on the ORF3 gene, all PEDV isolates could be divided into three groups namely: G1, G2, and G3 (Fig. 3). Notably, all Japanese field isolates clustered closely with US isolates and recent South Korea isolates, forming a separate sub-cluster within G1. On the other hand, the vaccine strains, P5-V and 96-P4C6, which have been used to prevent PEDV infection in Japan, were found within G3, which are therefore genetically distant from prevailing field isolates.

DNA sequence homology of 99.6‒100 % was observed between ORF3 genes of recent Japanese isolates that had the highest DNA identity (99.4‒100 %) with the aforementioned strains from the USA and South Korea. In particular, the common isolate 14JM-01 representing 21 PEDV samples from the 5 prefectures in Japan, had 100 % ORF3 gene homology with the majority of US strains (USA/Iowa/18984/2013, USA/Iowa/16465/2013, ISU13-22038-IA-passage9, IA1, IA2, OH1414, OH851, USA/Colorado/2013, MN, Iowa106, and ISU13-22038-IA-homogenate). In contrast, the ORF3 genes of the two vaccine strains, with exception of the identified unique deletions, were found to have low (95.8‒97.8 %) nucleotide identity with field Japanese isolates.

## Discussion

PED has been observed in Japan since the 1980s and a number of attenuated PED vaccines have been developed and used on pig farms to prevent this disease, particularly since late 2013 when PED has reemerged in Japan. However, PEDV infections still spread rapidly and are commonly observed on PED-vaccinated farms, leading to the loss of high numbers of pigs. As a result, the genetic characteristics and origins of prevailing PEDVs in Japan, the efficacy of PEDV vaccines being used in Japan in protecting previously well pigs from prevailing PEDVs, and the genetic differences between vaccine strains and field PEDV isolates remain critically important questions that have yet to be fully elucidated. We therefore performed this study to address these important issues regarding PED.

In this study, 88.5 % (77 of 87) of pig farms in six prefectures (Miyazaki, Kagoshima, Aichi, Hokkaido, Aomori, and Akita) were confirmed as infected with PEDV, and 72.5 % (148 of 204) of samples were found to be positive for PEDV. This result demonstrates a high prevalence of PEDV infection in Japanese pig herds.

The PEDV spike glycoprotein has a high degree of variability and contains several epitopes (Chang et al. 2002; Sun et al. 2008). Among these epitope sites, the COE domain (aa 499–638) is an important region capable of inducing PEDV-neutralizing antibodies (Chang et al. 2002). In comparison with vaccine strains (P-5V and 96P4C6) commonly used in Japan, all the field isolates were found to have 3–7 different residues in the COE domain, particularly at these 3 positions (517, 549, and 594). Notably, the amino acid at these three sites were all serine, one of few major amino acids that are capable of generating new O-linked glycosylation or phosphorylation. Netphos 2.0 server (http://www.cbs.dtu.dk/services/NetPhos) and NetPhosBac 1.0 Server (http://www.cbs.dtu.dk/services/NetPhosBac-1.0) were used for prediction of phosphorylation site. The result showed that phosphorylation was generated from serine residues at the position 517 and 549 of the field Japanese isolates. On the other hand, using BepiPred 1.0 Server (http://www.cbs.dtu.dk/services/BepiPred) to predict the location of linear B cell epitopes, no remarkable difference between the field PEDV isolates and the vaccine strains were found. Therefore, further research is needed to determine whether these amino acid differences may affect the antigenicity of prevailing PEDV isolates and consequently influence the efficacy of the vaccines currently used on Japanese pig farms.

In May 2013, PEDV was detected for the first time in the United States. Subsequently, US strain-like PEDVs were reported in South Korea in late 2013 (Lee and Lee 2014; Lee et al. 2014), and Germany in May 2014 (Hanke et al. 2015). The result of phylogenetic and genetic analysis of the partial S gene demonstrated that prevailing Japanese PEDV isolates had been previously unreported in Japan, shared the greatest similarity with US strain-like strains, and may have been introduced into Japan via unknown routes.

To date, two distinct PEDV strain types have been identified in US: the highly virulent US PEDV (US prototypes) (Stevenson et al. 2013) and the S INDEL PEDV variant, which contains insertions and deletions in the N-terminal region of the S protein, reported to cause milder disease in the field (Vlasova et al. 2014; Lee et al. 2014). Interestingly, isolate 14JM-140 was grouped in the same cluster as the S INDEL variant (OH851, IOW106, KNU-1406) with 100 % nucleotide identity observed between these strains. Clinical signs recorded on PEDV-infected farms demonstrated that only 24 piglets (out of a total of 400 pigs on the farm) had disease manifesting as diarrhea, with 4 piglets dying at the time of PED onset. This finding suggests the Japanese field isolate 14JM-140 may, in fact, be the S INDEL PEDV variant. This PEDV variant is prevalent and associated with low morbidity and mortality in PED outbreaks in Japan, although more extensive genome sequencing is required to clarify this finding.

ORF3 is an accessory gene thought to influence virulence and cell culture adaptation and has been used as a viral target in attempts to reduce PEDV virulence. Generally, ORF3 has utility as a valuable tool in the study of PEDV molecular epidemiology and for differentiating between field and vaccine-derived isolates. Our study revealed that the ORF3 gene of the Japanese vaccine strains, P5-V and 96P4C6, have unique deletions (49 and 4nt, respectively) that can lead to reading frame-shift and coding of truncated polypeptides. This genetic characteristic can be used to differentiate between field and attenuated-derived vaccine PEDV. Moreover, the finding of 49nt deletion in ORF3 gene of P5-V in this study is different from the result of a previous study (Park et al. 2008) in which authors reported that P5-V have 51nt deletion in ORF3 gene. This difference may be due to the genetic variation of P5-V during the time of the study. The results of the present study demonstrated that no deletion were observed in the Japanese field isolates, suggesting that they are not vaccine-related. Phylogenetic analysis further demonstrated that Japanese field isolates had greatest genetic similarity with US strains.

## Conclusions

This study demonstrated a high detection rate of PEDV on pig farms in Japan. All recent Japanese PEDV isolates were found to have previously unreported genotypes that differed from Japanese strains prior to 2013 as well as vaccine strains currently being used in Japan and had the greatest genetic similarity with US isolates, as compared with other countries. These findings suggest that prevailing PEDV isolates may have been introduced into Japan from overseas. The distant genetic relationship and amino acid differences in the neutralizing epitope COE domain between recent PEDV isolates and the vaccine strains may be responsible for unsuccessful PED control in Japan. Therefore, the development of new vaccines with greater protection against PEDV outbreaks in Japan is required.

## Methods

### Sample collection

A total of 204 samples were collected from suckling pigs, weaned pigs, and sows at 87 pig farms (farrow-to-finish and farrow-to-wean) experiencing acute diarrhea from six prefectures from north to south of Japan between December 2013 and October 2014. The number of samples from each prefecture was as follows: Miyazaki (n = 134), Kagoshima (n = 14), Aichi (n = 28), Akita (n = 3), Hokkaido (n = 10), Aomori (n = 15). One to 10 fecal samples, intestinal samples, or intestinal contents, were obtained from each outbreak of diarrhea. Fecal samples were taken from animals showing signs of diarrhea at the time of collection. Samples of intestine and intestinal content were collected from animals that have died due to severe diarrhea within 3 h. All the samples were temporarily preserved in ice boxes at time of collection, kept in icebox containing dry ice during transportation, and stored at freezer (−70 °C) when it arrived at the Laboratory of Veterinary Pathology, Univeristy of Miyazaki, using cryogenic freezing systems. Samples of two vaccine strains, P-5V (produced by Nisseiken Co., Ltd) and 96-P4C6 (produced by Kaketsuken Co., Ltd), were collected from commercial vaccine bottles used in pig farms in Japan.

### RNA isolation

Specimens from sick pigs were homogenized and diluted five times in Dulbecco’s Modified Eagle’s Medium with a low concentration of glucose. Samples were then centrifuged at 5000 rpm for 10 min at 4 °C. Supernatants were stored and subsequently used for RNA extraction. For each PEDV sample, total RNA was extracted from 100 to 300 µL aliquots of supernatant using ReliaPrep™ RNA Cell Miniprep kits (Promega Corpoation, WI, USA) in accordance with the manufacturer’s instructions.

### PEDV detection

The presence of PEDV in samples was detected by reverse transcription polymerase chain reaction (RT-PCR) using a previously published primer pair (Kim et al. 2001). Briefly, nucleotide strands of these primers are 5′-TTCTGAGTCACGAACAGCCA-3′ (P1, forward), 5′-CATATGCAGCCTGCTCTGAA-3′ (P2, reverse). The size of amplified product was 651 bp. The amplified genomic region of the S gene contains the neutralizing epitope region, CO-26K equivalent (COE) (Chang et al. 2002).

One tube RT-PCR reaction was performed using AccessQuick™ RT-PCR System kits (Promega Corpoation, WI, USA). Exactly, 4 µL of RNA template was mixed with a reaction mixture, which contained 12.5 µL of AccessQuickTM Master Mix (2×), 0.5 µL of each specific primer (10 µM), and 0.5 µL of AMV reverse transcriptase (5 u/µL). Then, 7 µL of nuclease-free water was added to reach the total volume reaction of 25 µL. The RT-PCR reaction were done using Takara PCR Thermal cycler (Japan). Following a reverse transcription step of 45 °C for 45 min and an incubation step of 94 °C for 2 min, 35 cycles were performed as follows: 94 °C for 30 s, 53 °C for 30 s, and 72 °C for 1 min. Cycles were followed by a terminal 10 min extension step at 72 °C. The last stage was preserving the PCR products at 4 °C. The RT-PCR products were visualized by electrophoresis in a 1.5 % agarose gel containing Ethidium Bromide.

### Amplification of the partial S gene and ORF3 gene

A primer pair was designed for amplifying the full ORF3 gene of PEDV with the following sequences: forward primer (ORF3-F), 5′-GTCCTAGACTTCAACCTTACGAAG-3′; and reverse primer (ORF3-R), 5′-AACTACTAGACCATTATCATTCAC-3′. The predicted size of the ORF3 PCR product was 740 bp. For the application of the partial S gene and the ORF3 gene of PEDV, RT was first performed using random primers and OligodT primers from Reverse Transcription System Kits (Promega, Madison, WI, USA). Complimentary DNA was immediately used to amplify the partial S gene (primer pair P1/P2) and ORF3 gene (primer pair ORF3-F/ORF3/R) using GoTaq^®^ Green Master Mix Kits (Promega, Madison, WI, USA) under the following condition: denaturation at 94 °C for 2 min; 35 cycles of denaturation at 94 °C for 30 s, annealing at 53 °C for 30 s, and extension at 72 °C for 1 min. PCR products were purified using FastGene Gel/PCR Extraction Kits (NIPPON Genetics Co., Ltd, Japan), according to the protocol of the commercial kit’s instruction.

### Sequencing

All sequencing reactions were carried out in duplicate and sequences were determined in both direction with BigDye^®^ Terminator v3.1 Cycle Sequencing Kits and an ABI PRISM 3130xl Genetic Analyzers (Applied Biosystems, CA, USA). The resultant nucleotide sequences were deposited in GenBank under the following accession numbers: KT968486-KT968518. Nucleotide and deduced amino acid sequences were edited, aligned (MUSCLE algorithm), and analyzed using BioEdit version 7.2.5 and molecular evolutionary genetics analysis (MEGA) software version 6.0 (Tamura et al. 2013). Phylogenetic trees based on the nucleotide sequences of the partial S gene and ORF3 gene were constructed with maximum likelihood method using Hasegawa-Kishino-Yano substitution model with discrete Gamma distribution, and bootstrap tests of 1000 replicates in the MEGA v.6 program.
